# Personality dimensions of schizophrenia patients compared to control subjects by gender and the relationship with illness severity

**DOI:** 10.1186/1471-244X-14-151

**Published:** 2014-05-24

**Authors:** Carmen Miralles, Yolanda Alonso, Begoña Verge, Sònia Setó, Ana M Gaviria, Lorena Moreno, María J Cortés, Alfonso Gutiérrez-Zotes, Elisabet Vilella, Lourdes Martorell

**Affiliations:** 1 Unitat de Psiquiatria, Hospital Universitari Psiquiàtric Institut Pere Mata, IISPV, Universitat Rovira i Virgili, CIBERSAM, C/ SantLlorenç, 21, 43201 Reus, Spain

**Keywords:** Schizophrenia, Personality dimensions, TCI-R, Gender, Illness severity

## Abstract

**Background:**

Personality traits and schizophrenia present gender differences; however, gender has not been considered in most studies on personality and schizophrenia. This study aims to identify the different personality dimensions of schizophrenia patients and healthy control subjects by gender and to explore the relationship between personality dimensions and illness severity variables by analyzing data for males and females separately.

**Methods:**

Temperament and Character Inventory-Revised dimensions were compared by gender between 161 schizophrenia patients and 214 healthy controls from a population-based sample using independent *t*-tests. We then investigated whether personality dimensions are related to illness severity variables using correlation analyses and bivariate logistic regression, also by gender.

**Results:**

The patients had significantly higher scores for harm avoidance (HA) and self-transcendence (ST) and lower scores for reward dependence (RD), cooperativeness (C), and self-directedness (SD) than the controls. Similar results were obtained when the sample was stratified by gender, however the differences were higher and more significant for HA among males and for RD among females. The number of admissions to a psychiatric hospital positively correlated with novelty seeking (NS) in males and negatively with SD in females. In males, SD and ST negatively correlated with the number of suicide attempts.

**Conclusions:**

Male and female patients present difficulties for regulating and adapting behavior to achieve goals (SD) and for identifying and accepting others (C), as well as a great sense of spirituality and universe identification (ST). However, male patients are more characterized by being fearful, doubtful and easily fatigued (HA), while female patients are characterized by presenting difficulties maintaining and pursuing associated reward behaviors (RD). Furthermore, male and female patients who are frequently admitted to psychiatric hospitals and male patients who attempt suicide should be evaluated regarding their personality dimensions. Future studies assessing the relationship between personality dimensions and the clinical features of schizophrenia should consider gender differences.

## Background

Personality is considered an important aspect of schizophrenia; it may influence symptom expression, patient insight, neurocognitive functioning, social functioning, and self-reported quality of life [1-3], and some premorbid personality traits may be related to an earlier presentation of disease [4,5]. Moreover, an overall prevalence rate of 39.5% for personality disorders has been reported among patients with psychotic disorders, although this prevalence rate does not differ from that for other severe mental illnesses [6]. Therefore, there is increasing interest in assessing the relationship between personality traits and the clinical features of psychotic disorders. Additionally, one major research focus is the use of personality traits to identify a specific profile associated with the risk of schizophrenia within the general population.

Historically, personality has been conceptualized through either categorical or dimensional models. Categorical models are based on the presence or absence of specific symptoms, while dimensional models assume a continuum between normality and abnormality and are thus the models of choice for assessing personality at least in control populations. Before the description of The Personality Inventory for DSM-5 (PID-5), there were many dimensional models of personality; some focused more on the characterization of normal personalities, whereas others were based on a dimensional reorganization of the diagnostic criteria for personality disorders. However, most of these models could be readily integrated within a common hierarchical structure [7]. The Temperament and Character Inventory (TCI) [8] is based on a psychobiological model with four temperament dimensions, including novelty seeking (NS), harm avoidance (HA), reward dependence (RD), and persistence (P), and three character dimensions, which are self-directedness (SD), cooperativeness (C), and self-transcendence (ST). NS reflects a tendency toward exploratory activity in response to novelty, impulsive decision making and active avoidance of monotony or frustration; HA is characterized by shyness, passive-avoidant behavior, rapid fatigability and worry in anticipation of possible danger; RD is the tendency to respond markedly to signals of reward and is manifest in social attachment and dependence; P is defined as perseverance despite frustration or fatigue; SD refers to self-determination, that is, the ability to control, regulate, and adapt behaviors to define, set, and pursue meaningful goals; C concerns the identification and acceptance of other people and reveals the tendency toward social tolerance, empathy, and compassion; and last, ST is associated with spirituality and with the concept of considering oneself an integral part of the universe. Studies conducted with the TCI have identified differences in both temperament and character between schizophrenia patients and control individuals. Most studies have observed that patients score significantly higher in HA and ST and significantly lower in SD and C compared with healthy controls, whereas the results for NS, RD, and P are inconclusive [9]. Regarding temperament, higher HA scores have been widely observed, and several authors have proposed that HA may be a genetic vulnerability marker for developing schizophrenia [10-12]. Regarding the character dimensions of the TCI, high ST and low SD and C scores have been associated with schizophrenia [9]. This same character profile was previously described as a schizotypal or disorganized configuration [8].

Schizophrenia research suggests that the clinical presentation of the disease generally favors females because females appear to have a better disease course than males, as they present better remission rates, lower relapse rates, and a lower risk of being admitted to the hospital than males. Additionally, males consistently present more negative symptoms, an earlier disease onset, and poorer premorbid functioning and social functioning [13,14], while females have superior mentalizing abilities [15]. Regarding personality, gender differences have also been identified in the general population when analyzing the temperament dimensions of the TCI, with females scoring higher for HA and RD [16]. However, in studies comparing schizophrenia patients and control subjects, if gender is included in the analysis, it is generally treated as a covariate. Only one study has presented independent analyses comparing male and female schizophrenia patients with a control population, but the authors did not differentiate gender in the control group [17]. Moreover, in a recent meta-analysis of personality traits and schizophrenia, gender was identified as a significant moderator of the effect size for HA, which was positively correlated with the proportion of males in the samples [9]. Table 1 shows the studies that have compared personality dimensions between schizophrenia patients and control subjects.

**Table 1:** Primary significant results of previous studies comparing personality dimensions between schizophrenia patients and control subjects

						**Personality dimensions**						
**Reference**		**Questionnaire**	**Study subjects**	**% of males**	**Population**	**NS**	**HA**	**RD**	**P**	**SD**	**C**	**ST**
[12]	Szoke et al.	TPQ	45 SCH	58	Paris,		⇑			X	X	X
			126 healthy controls	65	France							
[18]	Guillem et al.	TCI	52 SCH	71	Montreal,	⇓	⇑		⇓	⇓	⇓	
			25 healthy controls	52	Canada							
[19]	Ritsner and Susser	TPQ	90 SCH	86	Sha’ar Menashe,		⇑	⇓	X	X	X	X
			136 healthy controls	82	Israel							
[20]	Herran et al.	TPQ	59 SCH	53	Santander,		⇑	⇓		X	X	X
			43 healthy controls	56	Spain							
[21]	Boeker et al.	TCI	22 SCH	45	Magdeburg,				X	⇓	⇓	⇑
			22 healthy controls	55	Germany							
[22]	Calvó de Padilla et al.	TCI	11 SCH	62	Jujuy,			⇓		⇓	⇓	
			12 community controls	38	Argentina							
[17]	Hori et al. 2008	TCI	86 SCH	62	Tokyo,	⇓	⇑	⇓		⇓	⇓	⇑
			115 healthy controls	62	Japan							
[11]	Smith et al.	TCI	35 SCH	83	St. Louis,		⇑	⇓		⇓	⇓	⇑
			63 healthy controls	46	USA							
[23]	Gonzalez-Torres et al.	TCI	61 SCH (49%) and SCS (51%)	64	Bilbao,		⇑			⇓	⇓	⇑
			64 healthy controls	27	Spain							
[24]	Cortes et al.	TCI-R	29 SCH	86	Reus,		⇑	⇓		⇓		⇑
			188 controls	50	Spain							
[10]	Sim et al.	TCI	48 SCH (92%) and SCA (8%)	21	Seoul,		⇑	⇓	⇓	⇓	⇓	⇑
			106 healthy controls	41	South Korea							
[25]	Margetic et al.	TCI	120 SCH	58	Zagreb,	⇓	⇑			⇓		⇑
			120 healthy controls	58	Croatia							
[9]	Ohi et al.	TCI	99 SCH	55	Osaka,	⇓	⇑	⇓		⇓	⇓	⇑
			179 healthy controls	49	Japan							

TPQ: Tridimensional Personality Questionnaire; TCI: Temperament and Character Inventory; TCI-R: TCI-revised; SCH: schizophrenia; SCS: schizophrenia spectrum disorders; SCA: schizoaffective disorder.

X indicates dimensions not analyzed in the study.

Heterogeneity in personality traits may be related to clinical heterogeneity in schizophrenia and predict, for example, disease onset [4], suicide behavior [26-28] and hospital admission. To our knowledge, no studies have investigated the relationship between the number of psychiatric hospital admissions and personality traits in schizophrenia; however, hospital admission has been related to age of first hospital admission [29], stress management [30], neuroleptic treatment [31], gender, race [32], social support [33], and environmental conditions such as daily temperature range [34].

Despite extensive research attempting to identify a personality profile to differentiate schizophrenia patients from the general population, studies considering gender differences are scarce; however, males and females may have different personality profiles, and this may also extend to schizophrenia patients, for whom the appropriate therapeutic interventions may vary by gender. This study aimed to identify differences in personality dimensions between schizophrenia patients and healthy controls by analyzing males and females separately and to explore the relationship between personality and illness severity by gender.

## Methods

### Study design

This cross-sectional study was approved by the Clinical Research Ethics Committee of the Hospital Universitari Sant Joan de Reus. Informed, voluntary, written consent was obtained from the patients and controls after a thorough explanation of the procedures, in accordance with the Declaration of Helsinki.

Clinical information and demographic data were obtained through semistructured interviews with the patients, the patient’s relatives, and the controls. In patients, clinical information regarding age of disease onset, number of suicide attempts, and number of admissions to a psychiatric hospital were also obtained from the patient’s clinical records, as shown in Table 2.

**Table 2:** Characteristics of schizophrenia patients

	**Male**	**Female**	**P-value**
Number of patients	110	51	
Age (mean, SD)	36 (10.3)	40 (10.0)	0.013
Age of onset (median, range)	20 (12–39)	25 (13–48)	0.057
Psychiatric hospital admissions (median, range)	6 (1–24)	4 (0–20)	0.091
Suicide attempts (median, range)	0 (0–6)	0 (0–5)	0.674
Years of evolution of disease (median, range)	13 (0–45)	11 (0–49)	0.578
Marital status (N, percentage)			
Single	95 (87%)	32 (62%)	0.002
Married or paired	7 (6%)	12 (23%)	
Separated or divorced	8 (7%)	5 (11%)	
Widowed	0 (0%)	2 (4%)	
Drug abuse (N, percentage)	81 (74%)	33 (65%)	0.246

All patients were diagnosed with schizophrenia according to the DSM-IV criteria, either by clinical criteria or by the Schedules for Clinical Assessment in Neuropsychiatry interview (SCAN) [35]. For patients who had been admitted to the psychiatric hospital for more than six months, we considered that schizophrenia diagnoses could be properly established by independent clinical interviews with two experienced psychiatrists. However, for patients who were admitted for less than six months, including those who had their first contact with our hospital, we considered that schizophrenia diagnoses should be confirmed by the SCAN. The control group, obtained from a population-based sample, was composed of mentally healthy subjects selected through medical interviews and by screening with the 28-item scaled version of the Goldberg General Health Questionnaire (GHQ) [36,37].

### Participants

Patients were recruited from the Hospital Universitari Psiquiàtric Institut Pere Mata in Reus, Catalonia, Spain. Inclusion criteria consisted of a diagnosis of schizophrenia, clinical stability (symptoms could be present, but to a lesser extent than in the acute phase), age between 18 and 70 years old, white, parents born in Spain, no familial relationship with other study participants, and the ability to understand the nature of the study and to complete the questionnaire. Patients who were admitted to the hospital between 2009 and 2011 and fulfilled the inclusion criteria were invited to participate in the study. The control group was selected from a population-based sample between 18 and 75 years old (N = 800) that was randomly selected from the electoral rolls in three municipalities of a geographic region matching that of the patients. The inclusion criteria were Caucasian origin with parents and grandparents born in Spain, the capability of understanding the nature of the study, the absence of serious diseases that prevented participation, and in females, the absence of pregnancy or having given birth in the last 6 months. We selected unrelated participants (81 females and 133 males) who were 19 to 66 years old, had no antecedents of psychiatric illness, scored less than 7 on the 28-item GHQ, had valid data on the personality questionnaire, and did not differ statistically in age or gender from the patient group. A total of 161 schizophrenia patients and 214 control subjects participated in the study.

### Study variables

*Gender* was defined as male and female sex. *Age of disease onset* was established as the first occurrence of prominent psychotic symptoms and signs. *Number of suicide attempts* was defined as any voluntary, harmful behavior with a suicidal intent that the patients had experienced throughout life. *Number of admissions to a psychiatric hospital* was considered as admissions either in acute units or rehabilitation units the patients had experienced throughout life. *Personality dimensions* included novelty seeking (NS), harm avoidance (HA), reward dependence (RD), persistence (P), self-directedness (SD), cooperativeness (C), and self-transcendence (ST).

### Temperament and Character Inventory-Revised (TCI-R)

All participants completed the 240-item Spanish version of the TCI-R [38]. The items are scored using a five-point Likert scale ranging from 1 (strongly disagree) to 5 (strongly agree). The reliability and validity of The Spanish version of the TCI-R in the general population have been established [39]. The TCI-R was administered by clinical psychologists to the patients to avoid comprehension problems or attention bias during completion of the questionnaire and was self-administered for the control group.

### Statistics

Estimations of the power of this study for comparing two means at the 95% confidence intervals were calculated using Open Source Statistics for Public Health (Open Epi 2.3.1) at http://www.openepi.com.

The Student *t*-test, U Mann–Whitney test, or the *χ*^2^ test was used to compare the sociodemographic and clinical variables between the groups. The scores of all dimensions followed a normal distribution; therefore, we used a two-tailed *t*-test to compare the TCI-R scores for the dimensions of the patients and controls. We computed the Pearson correlation coefficient between the TCI-R dimensions and age. The number of admissions to a psychiatric hospital, number of suicide attempts, and age of disease onset were not normally distributed; therefore, a Spearman correlation analysis was conducted between the personality dimensions and illness severity variables. A bivariate logistic regression was used to predict the outcomes of variables related to illness severity based on the personality dimensions and other predictive variables contributing to the model. Two groups in the outcome variable were established: the 25^th^ percentile in the first group and the remaining in the second group.

The statistical significance level was set at p < 0.05. SPSS 19.0 for Windows was used to perform the analyses (Chicago, IL, USA).

## Results

### Case–control

**Figure 1:**
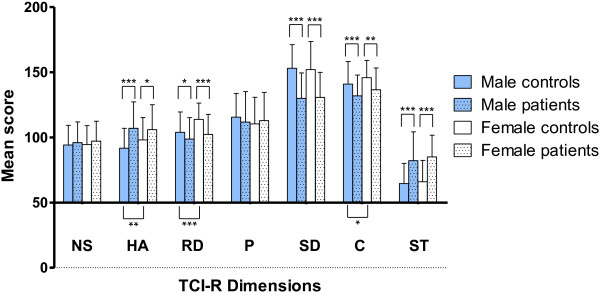
**TCI-R dimensions in schizophrenia patients and healthy controls by gender.** Mean scores in male controls (solid blue bars), male schizophrenia patients (dotted blue bars), female controls (solid white bars), and female schizophrenia patients (dotted white bars). Asterisks above the histogram bars indicate significant differences between patients and controls, and asterisks below the bars indicate differences between males and females in the control group (*p < 0.05; **p < 0.01; ***p < 0.001). Error bars represent standard deviations.

Patients had significantly higher scores for HA and ST and lower scores for RD, SD, and C than controls. However, because in the control group females had significantly higher scores than males for HA, RD, and C (Figure 1), we decided to stratify the sample by gender. Likewise, male and female patients had significantly higher scores for HA and ST and lower scores for RD, SD, and C than the male and female controls, respectively. However, the difference in the HA scores was significantly greater in the male group (difference between the means of 15.2 in males and 7.8 in females; p < 0.0001 and 0.015, respectively), whereas the difference in RD was significantly greater in the female group (difference between the means of 5.2 in males and 11.5 in females; p = 0.011 and <0.0001, respectively) (Figure 1 and Table 3). For detecting significant differences between the two means for each dimension in the male groups, the power calculations of the study were as follows: NS, 14.4%; HA, 100%; RD, 71.6%; P, 28.5%; SD, 100%; C, 98.9%; and ST, 100%. In the female groups, the power calculations were as follows: NS, 9.9%; HA, 66%; RD, 99.4%; P, 9.7%; SD, 100%; C, 92.1%; and ST, 100%.

**Table 3:** **Comparison of TCI-R dimensions between controls and schizophrenia patients by gender ( *t *-test)**

	**Males**				**Females**			
**TCI-R Dimension**	**Controls**	**SCH**	**md**	**P-value**	**Controls**	**SCH**	**md**	**P-value**
	(N = 133)	(N = 110)			(N = 81)	(N = 51)		
Novelty Seeking (NS)	94.2 ± 15.1	96.0 ± 15.9	1.8	0.368	95.4 ± 14.4	97.2 ± 15.2	1.8	0.496
Harm Avoidance (HA)	91.8 ± 15.2	107.0 ± 20.2	**15.2**	**<0.0001**	98.1 ± 17.2	105.9 ± 19.1	7.8	0.015
Reward Dependence (RD)	104.0 ± 15.5	98.8 ± 16.3	5.2	0.011	113.8 ± 12.5	102.3 ± 15.4	**11.5**	**<0.0001**
Persistence (P)	115.6 ± 18.2	111.8 ± 23.4	3.8	0.160	110.4 ± 20.4	112.9 ± 21.7	2.5	0.507
Self-directness (SD)	153.1 ± 18.1	130.0 ± 19.5	**23.1**	**<0.0001**	152.1 ± 21.5	130.7 ± 19.2	**21.4**	**<0.0001**
Cooperation (C)	141.0 ± 17.3	131.9 ± 16.0	9.1	**<0.0001**	145.9 ± 13.2	136.6 ± 16.7	9.3	**0.001**
Self-transcendence (ST)	64.6 ± 15.5	82.2 ± 22.1	**17.6**	**<0.0001**	65.9 ± 16.4	85.1 ± 16.6	**19.2**	**<0.0001**

md: differences between means; SCH: schizophrenia.

Data are given as the mean ± standard deviation. Mean differences above 10 points and p values lower or equal than 0.001 are highlighted in boldface.

Additionally, in the control group, we observed an inverse correlation of age with NS (r = −0.303, p < 0.001) and a direct correlation with ST (r = 0.320, p < 0.001) in males but not females. In the patient group, there were inverse correlations of age with NS (r = −0.332, p = 0.017), RD (r = −0.301, p = 0.032), and C (r = −0.290, p = 0.039) in females but not males.

### Illness severity

We investigated whether personality dimensions were correlated with variables associated with illness severity (number of psychiatric hospital admissions, number of suicide attempts, and age of disease onset). Distinct significant coefficient correlations were obtained based on gender. The number of psychiatric hospital admissions positively correlated with NS in males (r = 0.243; p = 0.012) and negatively correlated with SD in females (r = −0.308; p = 0.035). In males, but not females, the number of suicide attempts negatively correlated with SD (r = −0.222; p = 0.049) and ST (r = −0.322; p = 0.004). Table 4 shows that in the bivariate regression analysis, an increase in the NS score was directly related to an increase in the number of admissions in a psychiatric hospital in male but not female patients. In females, a decline in the SD score was directly related to an increase in the number of admissions to a psychiatric hospital. In male patients, increases in the SD and ST scores were related to a decrease in the number of suicide attempts. Age and years of evolution did not significantly contribute to the model and were therefore not included in the analysis. We want to note that male and female patients did not differ in either the median number of suicide attempts (median = 0 in both males and females; *U =* −0.421; p = 0.674; 25^th^ percentile = 0) or the years of evolution of the disease (median = 13 in males and 11 in females; *U =* −0556; p = 0.578; 25^th^ percentile = 4). The number of psychiatric hospital admissions was slightly higher in male patients than in female patients (median = 6 in males and 4 in females; *U =* −1.691; p = 0.091; 25^th^ percentile = 2), and the age of disease onset was lower in males than in females (median = 20 years in males and 25 years in females; *U =* −1.904; p = 0.057; 25^th^ percentile = 18). In male but not female patients, the number of psychiatric hospital admissions positively correlated with both the number of suicide attempts (r = 0.387; p < 0.001) and years of disease evolution (r = 0.315; p < 0.001) and was negatively correlated with the age of disease onset (r = −0.264; p < 0.01).

**Table 4:** Relationship between personality dimensions and severity of illness

**Gender**	**Dependent variable**	**Variables in the equation**	**Wald**	**OR (95% CI)**
Males	Number of admissions in a psychiatric hospital^a^	NS	8.838**	1.07 (1.02-1.12)
		Number of suicide attempts	3.560	2.74 (0.96-7.83)
		SD	9.692**	0.94 (0.90-0.98)
	Number of suicide attempts^b^	ST	9.581**	0.94 (0.91-0.98)
		Number of admissions in a psychiatric hospital	13.362***	1.37 (1.16-1.62)
Females	Number of admissions to a psychiatric hospital^c^	SD	4.380*	0.96 (0.93-0.99)

Binary logistic regression analyses assessing the relationship between personality dimensions and illness severity variables in male and female schizophrenia patients. OR: odds ratio; CI: confidence interval.

^a^R^2^ = 0.345; ^b^R^2^ = 0.526; ^c^R^2^= 0.140 (Nagelkerke).

*p < 0.05; **p < 0.01; ***p < 0.001.

## Discussion

### Case–control

This study compared a large number of schizophrenia patients and healthy controls, which facilitated an analysis by gender because male and female healthy controls differ statistically in HA, RD and C.

Compared with the controls, we observed that both male and female patients showed higher scores for harm avoidance (HA) and self-transcendence (ST) and lower scores for reward dependence (RD), self-directedness (SD), and cooperativeness (C). These data are consistent with most previously obtained results; however, no previous studies have analyzed males and females independently, although a case–control meta-analysis (384 patients and 656 controls) identified gender distribution as a significant moderator of the effect size for HA, which positively correlated with the proportion of males in the study sample [9]. We observed significant differences in HA and RD between patients and controls for both males and females; however, the differences were greater and more significant for HA among males and for RD among females. Thus, male patients who tend to be high in HA would be characterized by a greater tendency to be fearful, doubtful, easily fatigued and avoidant compared to healthy male subjects, whereas female patients who tend to be low in RD would be characterized by a low response to social pressure and difficulties maintaining and pursuing associated reward behaviors compared with healthy female subjects.

We did not identify differences in the personality dimensions between males and females in the patient group. Only one previous study has identified gender differences in HA [17]; however, the sample for this study was small, such that the results may be due to a type-I error in the statistical analyses. In the control group of the present study, females had significantly higher scores than males for HA, RD, and C. These gender differences, along with higher P scores among females, which were not observed in the present study, were previously identified in the Spanish population by our group [38]. Notably, the controls in the present study were obtained from a population-based sample and were determined to be mentally healthy by the GHQ. Furthermore, females also had higher scores than males for HA and RD in a meta-analysis of the temperament dimensions of the TCI, which was conducted in healthy populations; the authors of the meta-analysis suggested that these differences should be considered in future studies using the TCI [16].

### Illness severity

Interestingly, we found that illness severity was related to specific personality dimensions by gender. The number of suicide attempts was negatively correlated with SD and ST in male but not female schizophrenia patients. It has recently been reported that HA and P are higher whereas SD and C are lower in suicidal schizophrenia patients compared with nonsuicidal schizophrenia patients [26,28]. Additionally, low SD has been associated with current suicidal ideation, and low ST has been associated with lifetime suicide attempts in schizophrenia patients [27]. Therefore, although previous studies have shown that low SD scores are related to suicidal behavior, we found that the SD and ST scores were both related to suicidal behavior, but only in males. Hence, an increased number of suicide attempts in males could be related to their greater difficulties in regulating and adapting behavior and to a decreased sense of spirituality and universal identification. The study by Albayrak et al. (2012) [28] and our study were both conducted on schizophrenia patients and included similar sample sizes; however, Abayrak et al. used the true-false format of the TCI, whereas we used the 5-point Likert scale format of the TCI-R. Unfortunately, Abayrak et al. did not consider the effect of gender on the outcome variable.

The number of psychiatric hospital admissions was related to personality dimensions by gender because higher scores for NS were related to a higher number of admissions in males but not females, in whom a higher number of admissions was related to lower SD scores. In male patients, an increase in NS may be associated with an increased number of hospitalizations because of their impulsive decision making and active avoidance of monotony and frustration, which are indicators of low behavioral control. Interestingly, increased NS behavior has been associated with poor insight [40] and alcohol-related problems [41]. Moreover, schizophrenia patients with comorbid alcohol abuse show highly selective and greater NS than patients without a dual diagnosis [42]. Notwithstanding, in female schizophrenia patients, a decrease in SD may be associated with an increased number of admissions because of their absence of goals and poor fit or adaptation to their environment, which is related to poor social functioning.

It is widely accepted that males and females differ in personality traits because of both biological and sociocultural factors. In this sense, estrogen, testosterone, and oxytocin have been proposed to play roles in gender differences in the biological bases of personality. Interestingly, total testosterone and free testosterone levels have been positively associated with NS in a male sample [43], and genetic variation in the estrogen receptor alpha gene has been associated with HA in both males and females [44]. A large amount of evidence indicates that oxytocin is involved in social behavior, and recently, Tseng and colleagues have reported that in healthy participants, oxytocin blood levels are correlated with the total score and the interpersonal dysfunction dimensional scores of the Schizotypal Personality Questionnaire only in females [45]. Gender differences in social functioning, age of disease onset, course of illness, and other domains have also been reported for schizophrenia. Therefore, elucidating the role of gender in schizophrenia could help to better define the disease [13].

### Implications for future work

The present study has determined that gender is on the basis of differences in personality dimensions. This supports the consideration of gender in future studies of personality dimensions in psychiatric and non-psychiatric populations. Interestingly, personality has been related to other clinical conditions, such as cardiovascular risk [46] and depressive symptoms [47]. According to depressive symptoms, Kendler and Gardner have recently reported that personality and failures in interpersonal relationships play a stronger etiologic role in major depression for women than for men in a co-twin study that matched sisters and brothers on genetic and familial-environmental backgrounds [48]. Therefore, their study shed light on the etiologic pathways underlying major depression by considering the gender differences.

### Limitations of the study

Three important limitations of this study should be mentioned. The first limitation is that personality was assessed by the TCI-R and was not reviewed by other questionnaires. Notably, however, the personality dimensions measured by the TCI-R showed higher correlations with the Psychopathological personality Scales of the MMPI-2 PSY-5 in patients, supporting the convergent validity of the two constructs [39]. The second limitation of the study is that the symptomatology of patients was not assessed. Although patients were only evaluated when they were clinically stable and no prominent symptoms were present, we cannot rule out that psychotic symptoms were likely present and may have influenced the scores of personality dimensions as previously reported [11,12,18,24,49,50]. Finally, a third limitation is that psychometric TCI properties such as reliability, factor structure and temporal stability could not be taken into account because they have not been extensively evaluated in psychotic patients.

## Conclusions

The present study confirms differences in both temperament (HA and RD) and character (SD, C, and ST) between schizophrenia patients and healthy controls. Interestingly, the differences were higher and more significant for HA among males and for RD among females. Therefore, psychoeducational and psychosocial interventions should consider and be dependent upon the personality traits of schizophrenia patients by gender. Notably, personality traits are also related to illness severity by gender. Male and female patients who are frequently admitted to psychiatric hospitals and male patients who attempt suicide should be evaluated regarding their personality traits to focus efforts on educating and supporting patients presenting this high risk. From the viewpoint of prevention, exploring personality traits in male and female schizophrenia patients may be helpful in identifying those at high risk for hospital admission or attempted suicide.

## Competing interests

The author(s) declare that they have no competing interests.

## Author contributions

LMa developed the study design and conducted the statistical analyses with the advice of AMG. CM drafted the first version of the manuscript. YA collected the clinical data. CM, YA, BV, SS, LMo, and MJC recruited the patients, and LMa, AG-Z, and EV recruited the controls. All authors were involved in the analysis and interpretation of the data and revised the distinct versions of the manuscript. All authors read and approved the final version of the manuscript.

## Author information

CM is a clinical psychologist pursuing a Master’s Degree in Mental Health at the Universitat Rovira i Virgili. LM, AG-Z, and EV are PhD supervisors at this university.

## Pre-publication history

The pre-publication history for this paper can be accessed here:

http://www.biomedcentral.com/1471-244X/14/151/prepub

## References

[B1] Eklund M Hansson L Bengtsson-Tops A The influence of temperament and character on functioning and aspects of psychological health among people with schizophrenia Eur Psychiatry 2004 19 1 34 41 10.1016/j.eurpsy.2003.07.008 14969779

[B2] Jetha MK Goldberg JO Schmidt LA Temperament and its relation to social functioning in schizophrenia Int J Soc Psychiatry 2013 59 3 254 263 10.1177/0020764011433639 22271885

[B3] Lysaker PH Davis LW Social function in schizophrenia and schizoaffective disorder: associations with personality, symptoms and neurocognition Health Qual Life Outcomes 2004 2 15 10.1186/1477-7525-2-15 15025789 PMC398420

[B4] Remschmidt H Theisen F Early-onset schizophrenia Neuropsychobiology 2012 66 1 63 69 10.1159/000338548 22797279

[B5] Skokou M Gourzis P Demographic features and premorbid personality disorder traits in relation to age of onset and sex in paranoid schizophrenia Psychiatry Res 2014 215 3 554 559 10.1016/j.psychres.2014.01.018 24495576

[B6] Newton-Howes G Tyrer P North B Yang M The prevalence of personality disorder in schizophrenia and psychotic disorders: systematic review of rates and explanatory modelling Psychol Med 2008 38 8 1075 1082 18070369 10.1017/S0033291707002036

[B7] Widiger TA Integrating normal and abnormal personality structure: a proposal for DSM-V J Pers Disord 2011 25 3 338 363 10.1521/pedi.2011.25.3.338 21699396

[B8] Cloninger CR Svrakic DM Przybeck TR A psychobiological model of temperament and character Arch Gen Psychiatry 1993 50 12 975 990 10.1001/archpsyc.1993.01820240059008 8250684

[B9] Ohi K Hashimoto R Yasuda Y Fukumoto M Yamamori H Iwase M Kazui H Takeda M Personality traits and schizophrenia: evidence from a case–control study and meta-analysis Psychiatry Res 2012 198 1 7 11 10.1016/j.psychres.2011.12.018 22397918

[B10] Sim M Kim JH Yim SJ Cho SJ Kim SJ Increase in harm avoidance by genetic loading of schizophrenia Compr Psychiatry 2012 53 4 372 378 10.1016/j.comppsych.2011.05.004 21696715

[B11] Smith MJ Cloninger CR Harms MP Csernansky JG Temperament and character as schizophrenia-related endophenotypes in non-psychotic siblings Schizophr Res 2008 104 1–3 198 205 18718739 10.1016/j.schres.2008.06.025 PMC2565802

[B12] Szöke A Schürhoff F Ferhadian N Bellivier F Rouillon F Leboyer M Temperament in schizophrenia: a study of the tridimensional personality questionnaire (TPQ) Eur Psychiatry 2002 17 7 379 383 10.1016/S0924-9338(02)00700-9 12547303

[B13] Ochoa S Usall J Cobo J Labad X Kulkarni J Gender differences in schizophrenia and first-episode psychosis: a comprehensive literature review Schizophr Res Treatment 2012 2012 916198 22966451 10.1155/2012/916198 PMC3420456

[B14] Iniesta R Ochoa S Usall J Gender differences in service use in a sample of people with schizophrenia and other psychoses Schizophr Res Treatment 2012 2012 365452 22966434 10.1155/2012/365452 PMC3420527

[B15] Abu-Akel A Bo S Superior mentalizing abilities of female patients with schizophrenia Psychiatry Res 2013 210 3 794 799 10.1016/j.psychres.2013.09.013 24103909

[B16] Miettunen J Veijola J Lauronen E Kantojärvi L Joukamaa M Sex differences in Cloninger’s temperament dimensions–a meta-analysis Compr Psychiatry 2007 48 2 161 169 10.1016/j.comppsych.2006.10.007 17292707

[B17] Hori H Noguchi H Hashimoto R Nakabayashi T Saitoh O Murray RM Okabe S Kunugi H Personality in schizophrenia assessed with the Temperament and Character Inventory (TCI) Psychiatry Res 2008 160 2 175 183 10.1016/j.psychres.2007.05.015 18602163

[B18] Guillem F Bicu M Semkovska M Debruille JB The dimensional symptom structure of schizophrenia and its association with temperament and character Schizophr Res 2002 56 1–2 137 147 12084428 10.1016/s0920-9964(01)00257-2

[B19] Ritsner M Susser E Temperament types are associated with weak self-construct, elevated distress and emotion-oriented coping in schizophrenia: evidence for a complex vulnerability marker? Psychiatry Res 2004 128 3 219 228 10.1016/j.psychres.2004.06.007 15541778

[B20] Herrán A Sierra-Biddle D Cuesta MJ Sandoya M Vázquez-Barquero JL Can personality traits help us explain disability in chronic schizophrenia? Psychiatry Clin Neurosci 2006 60 5 538 545 10.1111/j.1440-1819.2006.01577.x 16958935

[B21] Boeker H Kleiser M Lehman D Jaenke L Bogerts B Northoff G Executive dysfunction, self, and ego pathology in schizophrenia: an exploratory study of neuropsychology and personality Compr Psychiatry 2006 47 1 7 19 10.1016/j.comppsych.2005.04.003 16324897

[B22] Calvó de Padilla M Padilla E González Alemán G Bourdieu M Guerrero G Strejilevich S Escobar JI Svrakic N Cloninger CR de Erausquin GA Temperament traits associated with risk of schizophrenia in an indigenous population of Argentina Schizophr Res 2006 83 2–3 299 302 16480854 10.1016/j.schres.2005.12.848

[B23] Gonzalez-Torres MA Inchausti L Ibáñez B Aristegui M Fernández-Rivas A Ruiz E Fernandez E Bayón C Temperament and character dimensions in patients with schizophrenia, relatives, and controls J Nerv Ment Dis 2009 197 7 514 519 10.1097/NMD.0b013e3181aacc1a 19597359

[B24] Cortés MJ Valero J Gutiérrez-Zotes JA Hernández A Moreno L Jariod M Martorell L Vilella E Labad A Psychopathology and personality traits in psychotic patients and their first-degree relatives Eur Psychiatry 2009 24 7 476 482 10.1016/j.eurpsy.2009.06.002 19699061

[B25] Margetić BA Jakovljević M Ivanec D Margetić B Temperament, character, and quality of life in patients with schizophrenia and their first-degree relatives Compr Psychiatry 2011 52 4 425 430 10.1016/j.comppsych.2010.08.007 21683179

[B26] Calati R Giegling I Rujescu D Hartmann AM Möller HJ de Ronchi D Serretti A Temperament and character of suicide attempters J Psychiatr Res 2008 42 11 938 945 10.1016/j.jpsychires.2007.10.006 18054960

[B27] Aukst Margetić B Jakovljević M Ivanec D Marčinko D Margetić B Jakšić N Current suicidality and previous suicidal attempts in patients with schizophrenia are associated with different dimensions of temperament and character Psychiatry Res 2012 200 2–3 120 125 22560807 10.1016/j.psychres.2012.04.016

[B28] Albayrak Y Ekinci O Cayköylü A Temperament and character personality profile in relation to suicide attempts in patients with schizophrenia Compr Psychiatry 2012 53 8 1130 1136 10.1016/j.comppsych.2012.04.007 22682677

[B29] Rabinowitz J Levine SZ Häfner H A population based elaboration of the role of age of onset on the course of schizophrenia Schizophr Res 2006 88 1–3 96 101 16962742 10.1016/j.schres.2006.07.007

[B30] Norman RMG Malla AK McLean TS McIntosh EM Neufeld RWJ Voruganti LP Cortese L An evaluation of a stress management program for individuals with schizophrenia Schizophr Res 2002 58 2–3 293 303 12409170 10.1016/s0920-9964(01)00371-1

[B31] Ascher-Svanum H Zhu B Faries D Ernst FR A comparison of olanzapine and risperidone on the risk of psychiatric hospitalization in the naturalistic treatment of patients with schizophrenia Ann Gen Hosp Psychiatry 2004 3 1 11 10.1186/1475-2832-3-11 15175112 PMC428579

[B32] Durbin A Rudoler D Durbin J Laporte A Callaghan RC Examining patient race and area predictors of inpatient admission for schizophrenia among hospital users in California J Immigr Minor Health 2013 in press 10.1007/s10903-013-9831-7 23636464

[B33] Simone C Carolin L Max S Reinhold K Associations between community characteristics and psychiatric admissions in an urban area Soc Psychiatry Psychiatr Epidemiol 2013 48 11 1797 1808 10.1007/s00127-013-0667-1 23460045

[B34] Sung T-I Chen M-J Lin C-Y Lung S-C Su H-J Relationship between mean daily ambient temperature range and hospital admissions for schizophrenia: results from a national cohort of psychiatric inpatients Sci Total Environ 2011 410–411 41 46 10.1016/j.scitotenv.2011.09.028 22018962

[B35] Vázquez-Barquero JL SCAN: Cuestionarios Para La Evaluación Clínica En Neuropsiquiatría 1993 Madrid: Meditor

[B36] Goldberg DP Hillier VF A scaled version of the General Health Questionnaire Psychol Med 1979 9 1 139 145 10.1017/S0033291700021644 424481

[B37] Lobo A Pérez-Echeverría MJ Artal J Validity of the scaled version of the General Health Questionnaire (GHQ-28) in a Spanish population Psychol Med 1986 16 1 135 140 10.1017/S0033291700002579 3961039

[B38] Gutiérrez-Zotes JA Bayón C Montserrat C Valero J Labad A Cloninger CR Fernández-Aranda F Temperament and Character Inventory Revised (TCI-R). Standardization and normative data in a general population sample Actas Esp Psiquiatr 2004 32 1 8 15 14963776

[B39] Gutiérrez-Zotes JA Cortés MJ Valero J Pena J Labad A Psychometric properties of the abbreviated Spanish version of TCI-R (TCI-140) and its relationship with the Psychopathological Personality Scales (MMPI-2 PSY-5) in patients Actas Esp Psiquiatr 2005 33 4 231 237 15999299

[B40] Ritsner MS Blumenkrantz H Predicting domain-specific insight of schizophrenia patients from symptomatology, multiple neurocognitive functions, and personality related traits Psychiatry Res 2007 149 1–3 59 69 17137634 10.1016/j.psychres.2006.01.002

[B41] Lange LA Kampov-Polevoy AB Garbutt JC Sweet liking and high novelty seeking: independent phenotypes associated with alcohol-related problems Alcohol Alcohol 2010 45 5 431 436 10.1093/alcalc/agq040 20663854

[B42] Kim JH Kim D Park S-H Lee HB Chung EK Novelty-seeking among schizophrenia patients with comorbid alcohol abuse J Nerv Ment Dis 2007 195 7 622 624 10.1097/NMD.0b013e318093f425 17632255

[B43] Määttänen I Jokela M Hintsa T Firtser S Kähönen M Jula A Raitakari OT Keltikangas-Järvinen L Testosterone and temperament traits in men: Longitudinal analysis Psychoneuroendocrinology 2013 38 10 2243 2248 10.1016/j.psyneuen.2013.04.009 24034714

[B44] Gade-Andavolu R Macmurray J Comings DE Calati R Chiesa A Serretti A Association between the estrogen receptor TA polymorphism and Harm avoidance Neurosci Lett 2009 467 2 155 158 10.1016/j.neulet.2009.10.028 19822194

[B45] Tseng HC Chi MH Lee L-T Tsai HC Lee IH Chen KC Yang YK Chen PS Sex-specific associations between plasma oxytocin levels and schizotypal personality features in healthy individuals J Psychiatr Res 2014 51 37 41 24411593 10.1016/j.jpsychires.2013.12.011

[B46] Rosenström T Jokela M Cloninger CR Hintsanen M Juonala M Raitakari O Viikari J Keltikangas-Järvinen L Associations between dimensional personality measures and preclinical atherosclerosis: the cardiovascular risk in Young Finns study J Psychosom Res 2012 72 5 336 343 10.1016/j.jpsychores.2012.02.003 22469275

[B47] Josefsson K Merjonen P Jokela M Pulkki-Råback L Keltikangas-Jarvinen L Personality profiles identify depressive symptoms over Ten years? A population-based study Depress Res Treat 2011 2011 431314 21876796 10.1155/2011/431314 PMC3162972

[B48] Kendler KS Gardner CO Sex differences in the pathways to major depression: a study of opposite-sex twin pairs Am J Psychiatry 2014 171 4 426 435 10.1176/appi.ajp.2013.13101375 24525762 PMC3972260

[B49] Poustka L Murray GK Jääskeläinen E Veijola J Jones P Isohanni M Miettunen J The influence of temperament on symptoms and functional outcome in people with psychosis in the Northern Finland 1966 Birth Cohort Eur Psychiatry 2010 25 1 26 32 10.1016/j.eurpsy.2009.09.006 19932601

[B50] Song YY Kang JI Kim SJ Lee MK Lee E An SK Temperament and character in individuals at ultra-high risk for psychosis and with first-episode schizophrenia: associations with psychopathology, psychosocial functioning, and aspects of psychological health Compr Psychiatry 2013 54 8 1161 1168 10.1016/j.comppsych.2013.05.015 23831396

